# Duplication of the urethra in an adult male presenting with scrotal fistula: a rare case report

**DOI:** 10.1093/jscr/rjab429

**Published:** 2021-09-29

**Authors:** Ghassen Tlili, Khaled ben Ahmed, Emir Acacha, Tlili Taghrid, Kamel Ktari, Majdoub Wiem, Mehdi Jaidane, Hamadi Saad

**Affiliations:** Department of Urology, Sahloul Teaching Hospital, Sousse, Tunisia; Department of Urology, Sidi Bouzid Hopsital, Sidi Bouzid, Tunisia; Department of Urology, Sahloul Teaching Hospital, Sousse, Tunisia; Department of Cytology, Farhat Hached Hospital, Sousse, Tunisia; Department of Urology, Fattouma Bourguiba Teaching Hospital, Monastir, Tunisia; Department of Cytology, Sahloul Teaching Hospital, Sousse, Tunisia; Department of Urology, Sahloul Teaching Hospital, Sousse, Tunisia; Department of Urology, Fattouma Bourguiba Teaching Hospital, Monastir, Tunisia

## Abstract

Urethral duplication is a rare congenital abnormality with varied clinical manifestations; to this day, <300 cases were reported in the literature. It is affecting mainly males and is nearly always diagnosed in childhood or adolescence. It may be complete or incomplete, and the most widely accepted classification of the different types of urethral duplication was developed by Effman *et al*. Herein, we present a rare case of urethral duplication revealed by scrotal fistula in an adult man. A duplication is an unusual form of *Y*-type duplication: it is an incomplete urethral duplication urethra opening on the scrotum without communication with the urethra or bladder.

## INTRODUCTION

**Figure 1:**
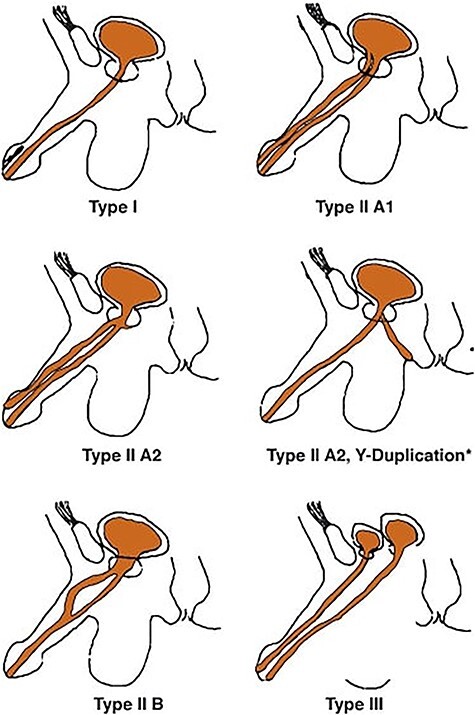
Classification of urethral duplications according to Effman.

Urethral duplication is a rare disease with varied clinical manifestations; to this day, <300 cases were reported in the literature [1]. It is affecting mainly males and is nearly always diagnosed in childhood or adolescence. It may be complete or incomplete, and the most widely accepted classification of the different types of urethral duplication was developed by Effman *et al*. (Fig. 1) [2]. Herein, we present a rare case of urethral duplication revealed by scrotal fistula in an adult man.

## CASE REPORT

**Figure 2:**
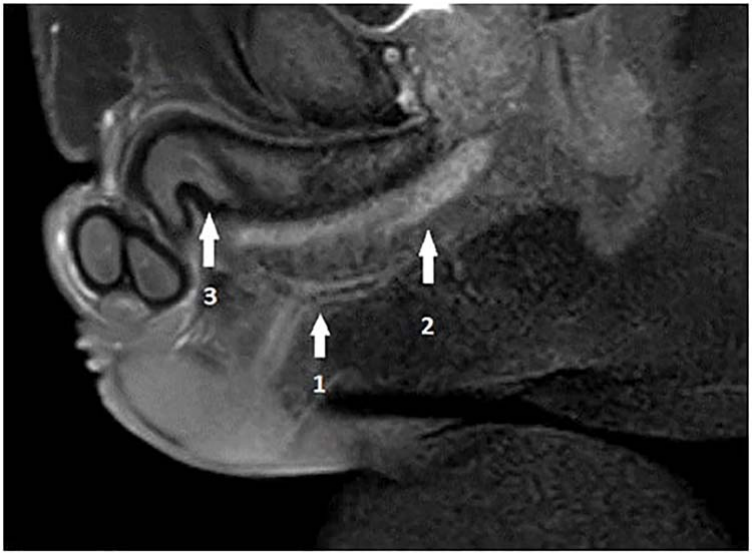
Pelvis MRI: horizontal one-eyed fistula (1) on the medial ligne above the corpus spongiosum (2), ending vertically with no collection and (3) corpus cavernosum.

**Figure 3:**
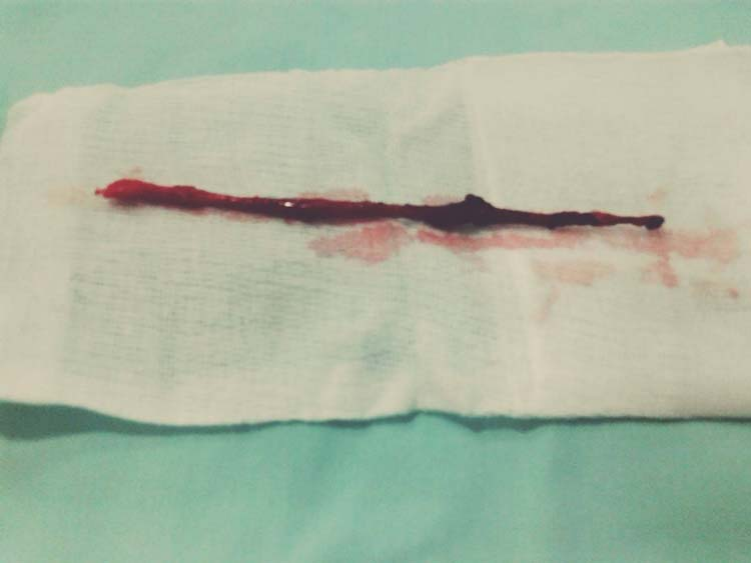
The blind channel after excision.

A 32-year-old male, with no previous major health problems, presented to our department complaining of scrotal fistula leakage of purulent fluid. The condition had started 6 months before. The physical examination revealed a productive scrotal fistula. He denied any dysuria or burning micturition or pyuria. His initial laboratory reports and urinalysis were unremarkable. A scrotal ultrasound revealed a thickening of the scrotal skin with no collection. Voiding cystourethrography was normal (no strictures). Magnetic resonance imaging (MRI) at that time showed a horizontal one-eyed fistula on the medial ligne above the corpus spongiosum, ending vertical with no collection (Fig. 2). The patient underwent an operative procedure, via a scrotal approach, the fistula was injected with methylene blue and a blind channel was excised from the surrounding tissues along his entire length. This channel measured 10.5 cm (Fig. 3). The diagnosis of urethral duplication had not been established at that time, but only on the pathology report.

**Figure 4:**
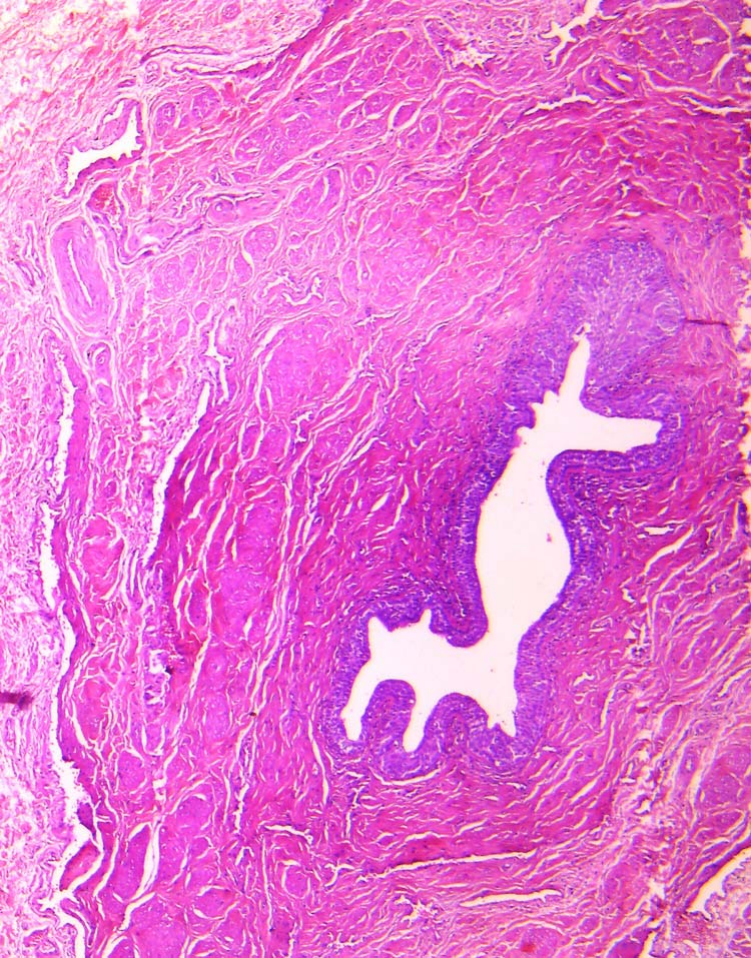
The lumen of the ureter covered by transitional epithelium.

The fistula (the channel) was lined with transitional and pseudostratified columnar epithelium with epidermic cover at the level of its scrotal extremity (Fig. 4). In retrospect, duplication is an unusual form of *Y*-type duplication. Incomplete urethral duplication with urethras opening on scrotum without communication with the urethra or bladder (Fig. 1). With 16-month follow-up, the patient is doing well. The patient remained asymptomatic and had returned to his usual life.

## DISCUSSION

The urethral duplication is a rare congenital abnormality <300 cases reported in the commune literature [1]. Embryogenesis is not well understood and various hypotheses exist, but none can explain all types of presentations; the common pathological process is supposed to result from an abnormal relationship between the lateral folds of the genital tubercle and the ventral end of the cloacal membrane, and the duplication commonly occurs in the sagittal plane with one urethra located ventrally and the other dorsally [3, 4]. Being a congenital, mostly the diagnosis was established in childhood or adolescence but nearly never in adult age [5], which makes our case one of few exceptions, with this anomaly being observed in an adult of 32 years of age with no other related congenital abnormality. The urethral duplication has multiple presentations, with a lack of specificity going from the asymptomatic, deformed penis, twin streams, urinary tract infection, symptoms of bladder outlet obstruction to various other signs like a renal failure as one of the worst [2–6]. In our case, the complaint was an anterior scrotal fistula with leakage of purulent fluid, and this type of complaint was rarely mentioned in the current literature.

Different types of urethral duplication classification exist, but the most accepted and the most widely used classification was developed by Effman *et al*., as this classification represents all clinical aspects of urethral duplication (Fig. 1) [2]. In retrospect, the duplication in our case was in an unusual form of *Y*-type duplication classification of Effman: a blind-ending accessory urethra (incomplete urethral duplication) opening on the surface of the scrotum. This new type of urethral duplication seems to be a variant between the Type AI and AII 2 or *Y*-type. Type AII 2 defines two urethras arising independently from a common bladder neck and states that the accessory urethra opens into the area perineum to rectum [7]; it is usually has a more functional ventral channel and a hypoplastic or stenotic dorsal (orthotopic) channel. However, when the ventral urethra is hypoplastic, the anomaly is classified as a congenital urethroperineal fistula. But for Wagner *et al*., all congenital urethroperineal fistulas are urethral duplications [8]. The type of urethral duplication of our patient has never been described. For the diagnosis, we performed a testicular ultrasound exam, a retrograde urethrogram and a pelvic MRI. In the literature, the main exam is the retrograde urethrogram. But in our case, it could not establish the diagnosis because of the particular anatomy of this type of *Y*-type duplication being none connected with the principal urethra.

Based on these radiological investigations, which were non relevant, we opted for the surgical exploration. The surgery reveals urethral duplication. Retrospectively, a sinogram and retrograde urethrogram should be performed simultaneously, and it may be found that the sinus was ending blindly in periurethral tissue and there was no communication with the urethra.

The urethral duplication may be associated with upper urinary tract anomalies, such as solitary kidney, hydronephrosis, multicystic dysplastic kidney and obstructive megaureter, and radiological investigation should be performed to evaluate the upper urinary tract [9, 10]. We did not find any other anomalies in our patient before beginning the treatment process. The treatment for duplicate urethra must be personalized and it depends on the patient’s symptoms and the type of anomaly. The *Y*-type duplications require more complex intervention than other kinds of repair [5]. The excision of the accessory channel is the most common surgical treatment, but alternative methods of sclerosis or fulguration of the accessory channel also have been reported, with risks of corporal thrombosis, fibrosis, impotency and incontinence which should be avoided [11]. In our case, the duplicated urethra was not functional. In fact, the surgical exploration was the key to the diagnosis of the urethral duplication, and we were only certain after the pathologist exam.

## CONCLUSION

Urethral duplication is a congenital anomaly with multiple anatomical presentations. Several theories about the etiology exist, but none can explain all types. There is also more than one rating available, and the Effmann classification is the most accepted, but no one has described this unusual *Y*-type of urethral duplication. The most specific radiological investigation for the diagnosis is the retrograde urethrogram in our case a simulated sinogram could help for the diagnosis. The treatment is surgical management and requires individualized planning and vast technical knowledge of reconstructive surgery.

## CONFLICT OF INTEREST STATEMENT

All authors declare no conflict of interrest.
